# Analytical Evaluation of Point-of-Care Finecare™ Procalcitonin Rapid Quantitative Test in Sepsis Population as Compared with Elecsys^®^ BRAHMS Procalcitonin Immunoassay

**DOI:** 10.3390/diagnostics14111080

**Published:** 2024-05-22

**Authors:** Mohd Zulfakar Mazlan, Wan Norlina Wan Azman, Najib Majdi Yaacob, Tan Say Koon, Nurul Khaiza Yahya

**Affiliations:** 1 Department of Anaesthesiology and Intensive Care, School of Medical Sciences, Universiti Sains Malaysia, Kubang Kerian 16150, Kelantan, Malaysia; 2 Department of Chemical Pathology, School of Medical Sciences, Universiti Sains Malaysia, Kubang Kerian 16150, Kelantan, Malaysia; dr_wannorlina@usm.my (W.N.W.A.); saykoon@usm.my (T.S.K.); 3 Biostatistics and Research Methodology Unit, School of Medical Sciences, Universiti Sains Malaysia, Kubang Kerian 16150, Kelantan, Malaysia; najibmy@usm.my; 4 Department of Immunology, School of Medical Sciences, Universiti Sains Malaysia, Kubang Kerian 16150, Kelantan, Malaysia; nuruliza@usm.my

**Keywords:** procalcitonin, sepsis, point of care, intensive care unit

## Abstract

The study compared two plasma procalcitonin (PCT) assays, the point of care (POC) Finecare™ Procalcitonin Rapid Quantitative Test and the Elecsys^®^ BRAHMS PCT immunoassay, in sepsis ICU patients. Forty-one plasma samples were analyzed, showing a strong correlation (r = 0.98) and no significant difference in PCT values. The mean POC PCT value was 4.46 ng/mL (SD 8.68), and for laboratory BRAHMS PCT, it was 4.67 ng/mL (SD 10.03). The study found a strong linear relationship between plasma POC PCT and laboratory BRAHMS PCT (r = 0.98). Different regression methods showed varying intercepts and slopes: Ordinary Least Squares had an intercept of 0.49 and a slope of 0.85; Deming regression showed an intercept of 0.43 and a slope of 0.86; Passing–Bablok regression showed an intercept of 0.02 and a slope of 1.08. Precision results for cut-offs of 0.5 ng/mL were a coefficient of variation (CV) of 5%, and for 2.5 ng/mL, the CV was 2.5%. The Pearson correlation coefficient (r) for linearity was ≥0.99. The study revealed no significant difference between the POC Finecare™ PCT and Elecsys^®^ BRAHMS PCT immunoassay in sepsis samples from ICU patients, supported by strong correlation, minimal bias, a consistent CV, and linearity.

## 1. Introduction

Procalcitonin (PCT) is a protein that was first discovered as a marker of bacterial infection in 1993 in a patient with extrathyroidal disease. In patients without disease, the normal level is 0.1 ng/mL. In the same year, it was found that procalcitonin levels were high (6–53 ng/mL) in children with bacterial infections and low (0.1–1.5 ng/mL) in viral infections [1]. PCT levels can be detected as early as 4 h, peak at 6 h, and then remain stable at a plateau between 8 and 24 h following the injection of E. coli endotoxin into healthy volunteers [2]. The most effective and commonly utilized cut-off value for PCT is 0.5 ng/mL, showing a sensitivity of 76% and a specificity of 69% [3]. The value of PCT for clinicians lies in its role as a biomarker for sepsis, aiding in antibiotic initiation, determining antibiotic duration, and guiding antibiotic cessation. Among these indications, the recommendation with the highest quality of evidence is for guiding antibiotic cessation, as demonstrated in the 2010 ProRATA trial [4]. The 2021 Surviving Sepsis Campaign guidelines also advocate for employing PCT to guide antibiotic duration in situations where determining the optimal duration of antibiotics is uncertain, suggesting its use in conjunction with clinical criteria rather than relying solely on clinical indicators [5].

Initiating antibiotics within one hour of septic shock and three hours in its absence is crucial [5]. PCT can aid clinicians in making rapid decisions when it is available. However, PCT testing such as Elecsys^®^ BRAHMS Procalcitonin immunoassay on laboratory analyzers such as the Cobas e411 requires more time compared to point-of-care (POC) PCT testing bedside in the intensive care unit (ICU). Hence, evaluating the accuracy of POC testing against laboratory immunoassay methods is crucial to maintaining acceptable accuracy. This approach aids clinicians in appropriately initiating and discontinuing antibiotics, thereby promoting antibiotic stewardship.

Examples of POC analyzers available in 2021 for PCT testing include the AQT90 FLEX (Radiometer, Copenhagen, Denmark), mLabs (Micropoint, Shenzhen, China), Finecare (Wondfo, Guangzhou, China), and Getein 1100 (Getein Biotech Inc., Nanjing, China) [6]. Studies indicate that the AQT90 FLEX PCT assay demonstrates good agreement and precision compared to the reference immunoassay [6]. A coefficient of variation (CV) of less than 5% was observed in both plasma and whole blood at 0.5 ng/mL and 2.0 ng/mL PCT levels [6]. However, the assay stability is only up to 8 months and requires conditions of 2–8 degrees for storage of the reagent [6].

Prior to the availability of POC PCT testing, the automated assay (Vidas Brahms PCT test with enzyme-linked fluorescent assay (ELFA) manufactured by bioMérieux, Marcy-l’Étoile, France) was used, starting in 2007 [7]. It was first approved in the United States of America in 2007 and gained further approval in 2016 for its use in assessing risk for mortality by serial PCT in 96 h [7]. The BRAHMS PCT-sensitive Kryptor^®^ test (Thermo Fisher Scientific, Waltham, MA, USA) with the Time-Resolved Amplified Cryptate Emission (TRACE) immunoassay was approved in 2008 [8]. Several other examples of automated immunoassay platforms for PCT testing and their mechanisms of immunoassay are available, including the Liaison BRAHMS PCT (DiaSorin, Saluggia, Italy) with two-site immunoluminometric assay, Roche BRAHMS PCT (Roche Diagnostics, Basel, Switzerland) with electrochemiluminescence immunoassay (ECLIA), and Siemens BRAHMS PCT (Siemens Healthineers, Erlangen, Germany) with one-step chemiluminescence immunoassay, Diazyme PCT assays (Diazyme Laboratories, Poway, CA, USA) with latex-enhanced immunotubidimetric assay [9]. These platforms offer efficient and reliable PCT testing, contributing to the management of various clinical conditions with varying reporting of bias [9].

The accuracy of PCT tests is significantly impacted by the quality of the assay. Assays employ different technologies, such as luminescence and turbidimetry, to measure PCT levels, and their reliability and precision directly affect measurement accuracy. High-quality assays ensure consistent and reproducible PCT detection, yielding reliable results. Conversely, lower-quality assays may introduce variability and errors, compromising the overall accuracy of PCT testing. A prior study demonstrated that the type of sample used can affect the precision of procalcitonin (PCT) testing [6]. Specifically, it found that whole blood samples had higher coefficients of variation (CVs), indicating lower precision. In contrast, the precision was better when using plasma samples for point-of-care PCT testing. Therefore, it is crucial to use high-quality POC assays to obtain accurate and clinically relevant PCT test results. The aim of the study was to evaluate the analytical performance of the Finecare POC PCT test manufactured by (Wondfo, Guangzhou, China) compared to the Elecsys^®^ BRAHMS PCT assay manufactured by (Roche Diagnostics, Mannheim, Germany) as a reference assay.

## 2. Materials and Methods

The study was approved by the Institutional Review Board (IRB) of the [USM Code: USM/JEPeM/21030239]. Informed consent was obtained from all patients or their legal representatives prior to enrolment in the study in 2023. The study employed a random sampling strategy to identify participants. Eligible patients were those admitted to the intensive care unit within the previous 24 to 48 h, carrying a diagnosis of sepsis, and receiving antibiotic treatment for sepsis management. Whole blood samples were collected from the in situ arterial line of each enrolled patient once only. Within 30 min of collection, the whole blood samples were tested by a laboratory technician using the Finecare™ POC PCT test to cover all significant PCT values in the range of 0.1–100 ng/mL.

The remaining portion of the same blood sample was centrifuged to obtain plasma. The generated plasma samples underwent further testing for PCT levels using both Finecare™ POC PCT and Elecsys^®^ BRAHMS PCT to cover all significant PCT values in the range of 0.02–100 ng/mL. Prior to testing, both PCT analyzers underwent quality control checks to ensure accurate results during the day of testing.

The PCT levels obtained from plasma samples using the Finecare™ POC PCT test were compared with the plasma Elecsys^®^ BRAHMS PCT results obtained from the Cobas e411 analyzer. The remaining plasma aliquot was kept at −20 degrees in a chemical pathology laboratory freezer for precision and linearity studies within 3 weeks. The mean time of mobilizing the samples to the participating room was less than 5 min. The laboratory room temperature was set at 18–24 degrees. Precision was assessed using two cut-off values: 0.5 ng/mL and 2.5 ng/mL, to determine the accuracy and reliability of the POCT Finecare system. Linearity testing was conducted using serum samples with low (0.155 ng/mL) and high (82.76 ng/mL) PCT values from the laboratory’s results to evaluate the performance of the POCT Finecare system across the PCT concentration range.

### 2.1. Overview of POC PCT Test Analyzer

#### 2.1.1. Finecare FIA Meter

The Finecare FIA meter is a compact benchtop device that utilizes immunofluorescence and is suitable for bedside use in the ICU. The analyzer and kit were manufactured by Wondfo, Guangzhou, China. It employs specialized fluorescent immunoassay technology for measuring biomarkers associated with infection, diabetes, oncology, cardiovascular disease, and kidney disease. The POC PCT assay utilized in this study is the Finecare™ PCT Rapid Quantitative Test, with Reagent Lot F21016807A7D. The test can be performed by any trained healthcare professional, with results available in 15 min. Each kit box contains 25 PCT test kits. Only 50 microliters of plasma/serum from centrifuged ethylenediaminetetraacetic acid (EDTA) or plain tubes, or 75 microliters of whole blood from a 3 mL EDTA bottle, are required for the test. The kit has a shelf life of 2 years for each batch and remains stable for 24 months at room temperature. The reportable range is 0.1 ng/mL to 100 ng/mL, with a CV of less than 10% reported by the manufacturer at the 0.5 ng/mL cut-off. The functional sensitivity is 0.1 ng/mL.

#### 2.1.2. Cobas e411

The Cobas e411 is a large benchtop analyzer used in laboratories for chemical pathology tests. It is a standard reference immunoassay laboratory analyzer capable of performing various tests on large sample groups cost-effectively. The Elecsys^®^ BRAHMS PCT assay used in this study was manufactured by Roche Diagnostics, Mannheim, Germany. The tests must be conducted by trained laboratory technicians, with samples pipetted and reagents stored at 2–8 degrees Celsius. To ensure cost-effectiveness, a minimum of 100 tests, including calibration, are required per day. The time for results to be ready depends on transportation and the arrival of the sample to the laboratory; in our center, we are still using human couriers for sample transportation, compared to other centers that utilize pneumatic tube systems. The reportable range is 0.02–100 ng/mL, with a functional sensitivity of 0.06 ng/mL and a CV of less than 5% at the 0.5 ng/mL cut-off. The reference assay used is the Elecsys^®^ BRAHMS PCT assay with Reagent Lot 63026904.

### 2.2. Statistical Analysis

In this study, descriptive statistics summarized the data, and histograms visualized its distribution. Outliers were identified and removed, and we recalculated descriptive statistics and created new histograms. Data distributions for both PCTs were analyzed separately. Correlation analysis determined variable relationships. A paired *t*-test compared means of paired samples. Ordinary Least Squares (OLS) regression analyzed linear relationships, plotting the OLS regression line. Deming regression and Passing–Bablok regression analyses were performed, plotting their respective lines. A comparison plot compared variables or datasets. Bland–Altman analysis assessed agreement between measurements. Mean and standard deviation (SD) of both measurements were calculated 17 times for CV, excluding the highest and lowest values for a total of 15 measurements for the final CV calculation. Linearity was analyzed by the Spearman correlation test.

### 2.3. Details of Method

#### 2.3.1. Accuracy

Accuracy was assessed using Ordinary Least Squares, Passing–Bablok linear regression, Deming regression, and Bland–Altman analysis of 41 samples with PCT levels, comparing the Finecare™ PCT Rapid Quantitative Test with the Elecsys^®^ BRAHMS PCT assay on the Cobas e411. OLS was used when both variables were error-free, Deming regression when error could exist in both, and Passing–Bablok to detect systematic differences. An intercept of zero or close to zero indicates no systematic differences between the methods, while a slope close to 1 indicates a strong relationship between them.

#### 2.3.2. Precision

In plasma, we assessed analytical precision by measuring within-run precision in two plasma samples, each tested with both PCT assays. Plasma samples with PCT concentrations of 0.5 ng/mL and 2.5 ng/mL, as tested by the Cobas e411, were selected for evaluation on the Finecare PCT analyzer, the Finecare Fia Meter III Plus (FS-205). Each plasma sample was tested 17 times, excluding the highest and lowest values, and the remaining 15 values were used to calculate the mean, standard deviation (SD), and coefficient of variation (CV). Analytical precision in whole blood was not evaluated because the Cobas assay was validated only for plasma/serum samples.

#### 2.3.3. Linearity

Linearity was assessed in plasma mix dilutions using pooled procalcitonin (PCT) samples at concentrations of 0.155 ng/mL (low) and 82.76 ng/mL (high), following the Cobas assay protocol. Dilutions were prepared by combining the samples with PCT-negative pooled plasma in a 6-sample dilution scheme of 200 microliters. Sample 1 represented the low PCT value, while sample 6 represented the high PCT value. Samples 2, 3, 4, and 5 consisted of various ratios of low and high PCT [0.8 low and 0.2 high for sample 2, 0.6 low and 0.4 high for sample 3, 0.4 low and 0.6 high for sample 4, and 0.2 low and 0.8 high for sample 5]. Theoretical PCT concentrations in the dilutions were calculated based on these ratios after averaging three readings, and the mean values were used for analysis. A correlation coefficient of 0.995 or higher between the calculated and actual results was considered acceptable.

## 3. Results

The study population was predominantly male, with a majority age of 51 years. A significant proportion, 68%, had acute kidney injury as evidenced by elevated creatinine levels. All patients included in the study exhibited elevated total white blood cell counts, confirming their sepsis diagnosis. Most of the study participants required mechanical ventilation, indicating the severity of their condition. Approximately 40% of the patients were in septic shock (Table 1).

The mean plasma POC Finecare™ PCT Rapid Quantitative Test value was 4.46 ng/mL (SD 8.68), and Elecsys^®^ BRAHMS PCT assay on the Cobas e411 was 4.67 ng/mL (SD 10.03) (Table 2).

The mean difference was −0.21 (95% CI −0.91 to 0.49), with a *p*-value of 0.556 (Table 3).

**Figure 1:**
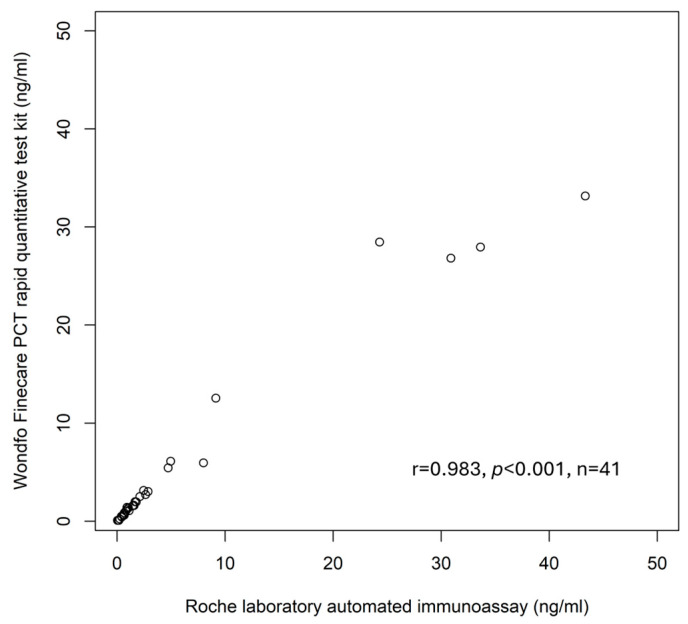
Scatter Plot.

The linear relationship between plasma POC PCT and plasma Elecsys^®^ BRAHMS PCT is 0.98 (Figure 1).

**Figure 2:**
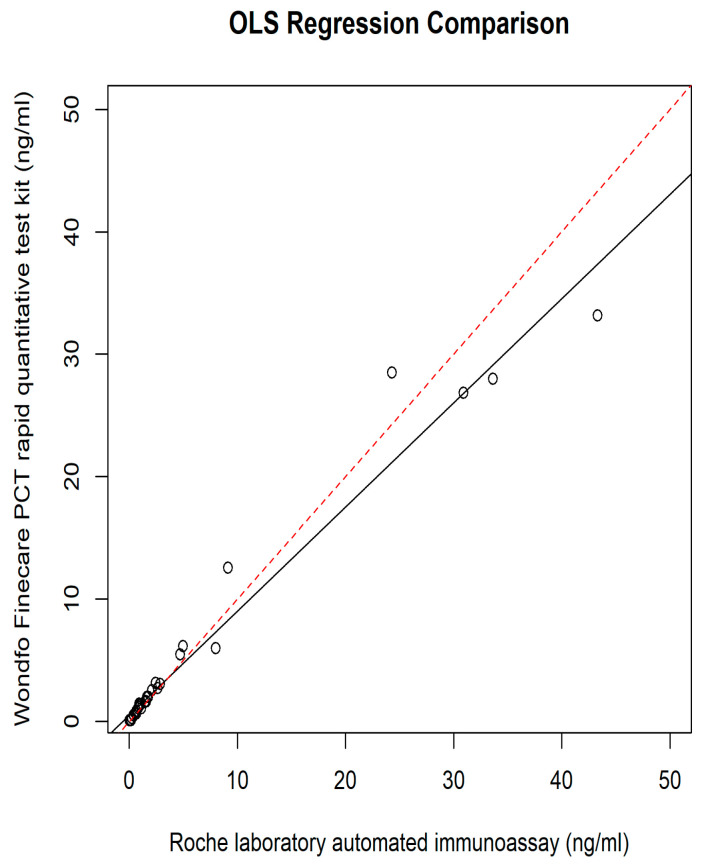
OLS regression.

**Figure 3:**
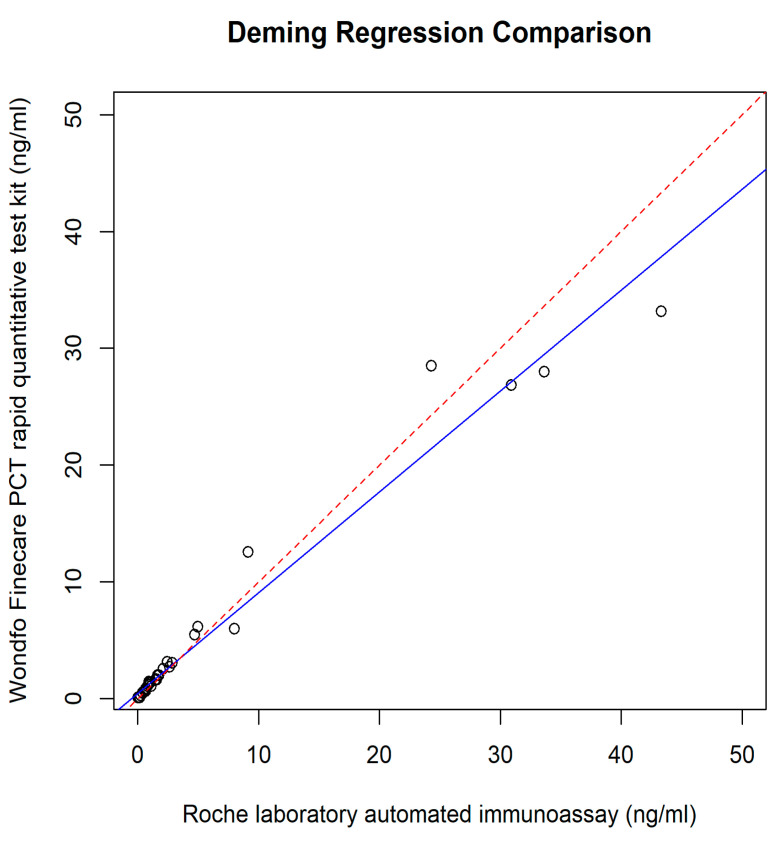
Deming regression.

**Figure 4:**
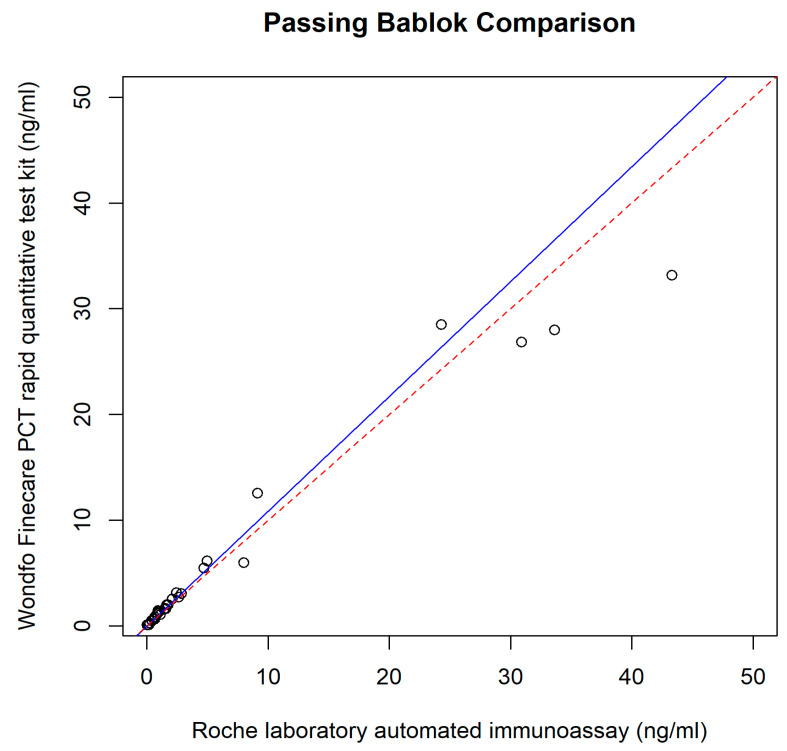
Passing–Bablok regression.

**Figure 5:**
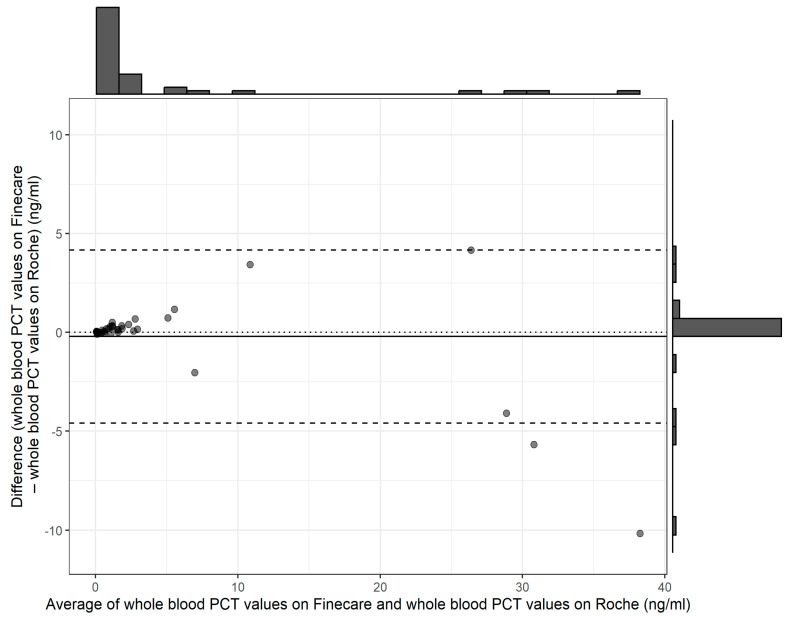
Baltman analysis.

The relationship between the POC PCT and plasma Elecsys^®^ BRAHMS PCT was assessed using different regression methods, which reveal intercepts {(95% confidence interval (CI)} and slopes (95% CI) for each method; they were as follows: Ordinary Least Squares had an intercept of 0.49 (−0.06 to 1.05) and a slope of 0.85 (0.80 to 0.90); Deming regression showed an intercept of 0.43 (−0.05 to 0.71) and a slope of 0.86 (0.78 to 1.15); Passing–Bablok regression revealed an intercept of 0.02 (−0.04 to 0.06) and a slope of 1.08 (0.94 to 1.18) (Figure 2, Figure 3, Figure 4 and Figure 5).

The repeated tests of plasma PCT after 3 weeks showed almost similar results on the analyzer; hence, the samples at the –25 Celsius temperatures were stable for 3 weeks. The precision results for the chosen cut-offs of 0.5 ng/mL, with a CV of 5%, and 2.5 ng/mL, with a CV of 2.5%, were obtained. The Pearson correlation coefficient (r) was found to be greater than or equal to 0.99 for linearity.

## 4. Discussion

This study focused on the correlation, precision, and linearity of the point-of-care Finecare™ PCT Rapid Quantitative Test in relation to the reference immunoassay, the Elecsys^®^ BRAHMS PCT assay performed by the Cobas e411 in the central diagnostic laboratory. The patient sampling was from mixed ICU patients diagnosed with sepsis and admitted to the ICU. Our institution is a teaching tertiary university hospital located on the east coast of Malaysia, with about 850 beds and a 15-bed mixed ICU unit. The total admissions to the mixed ICU are approximately 800 patients per year. Most laboratory investigations, including biomarker tests, were performed in the central diagnostic laboratory.

The laboratory immunoassay is expensive and takes approximately 12–24 h to deliver all the biochemical results, including the PCT results, to our institution. Furthermore, the laboratory immunoassay is subject to interference, for example, from antibodies, biotin, and the hook effect, which may cause falsely low or high results. The extended duration required for result availability can be attributed to several factors. Certain tests are not performed on a daily or routine basis; instead, their scheduling depends on the overall workload. Additionally, for reasons of cost-effectiveness, some tests are conducted in batches rather than individually, which can further prolong the turnaround time. Additionally, the delay in immunoassay results is also affected by the validation and interpretative comments provided by the respective pathologists, especially for hormonal tests, if there is a discrepancy from previous results or the patient’s clinical condition. One advantage of the point-of-care PCT testing used in our study is the longer expiration date of around 2 years, and since the kits come in boxes of 25, using some kits does not affect the remaining kits in the same box. The minimum purchase quantity is 25 test kits per box, which costs between USD 300 and 400 per box. In comparison, laboratory-based PCT testing is more expensive, with each box costing USD 1500–2000 but allowing for 100 tests to be performed. However, in practical usage, the number of tests that can be performed from a laboratory PCT kit depends on the daily testing volume and frequency. Each day, laboratory PCT testing requires calibration for every PCT test planned, so if a bulk order of 99 samples is received in one day, only one test is needed for calibration. But in the worst-case scenario of only one PCT test being sent to the lab per day for 50 days, only 50 tests can be performed because an additional test is used for daily calibration over those 50 days. Therefore, the minimum number of tests required is 25 tests within the 2-year expiration date for point-of-care testing, and 50 PCT tests within the expiration date per box of laboratory reagent.

This delay may not be conducive to prompt clinical decision-making, especially in sepsis cases. The delay is due to the overall process, starting from sample collection, sample delivery and checking, registration, sample processing, and waiting time for many samples to be processed at once. Additionally, sample results entry involves a few staff members. While this process is beneficial for quality control of the results, the longer time taken can affect decision-making for certain biomarkers that require prompt action by clinicians and consultants conducting the ward round in the morning. Studies have shown that mortality rates decrease when source control is managed within 6 h [10]. Therefore, it is crucial to have a reliable POC PCT test as an alternative to laboratory PCT tests, particularly in developing countries with limited budgets for additional laboratory hospital tests. Among the automated PCT immunoassay platforms (Liaison, Vidas, Cobas e601, Advia Centaur, Diazyme on Architect, Diazyme on Advia 2400, Diazyme on Cobas c501, and Diazyme on AU5800), agreement ranges from 83% to 98% for a 0.5 ng/mL cut-off and a 91% to 97% agreement for a cut-off of 2 ng/mL, compared to BRAHMAS Kryptor PCT (Thermo Fisher Scientific, Hennigsdorf, Germany) as a reference method [9]. Although for automated immunoassays performed in the laboratory, there is a presence of modest bias among the automated immunoassays, this can be overcome by continuous and systematic tracking of a patient’s health status, symptoms, and medical data over an extended period [9]. The automated immunoassays also reveal variability in the PCT results with various manufacturers. The variability ranges from −13% to 49% between the Abbott, Beckmann, Biomerieux, DiaSys, Roche, Siemens, and Thermo-BRAHMS participants [11]. The variations in results could be due to various aspects such as instrument calibration, sample quality, and technical factors. The technologies used also differ, such Time-Resolved Amplification of Cryptate Emission (TRACE), chemiluminescence immunoassay and electrochemiluminescence immunoassay (CLIA/E-CLIA), enzyme-linked fluorescent assay (ELFA), and Particle-Enhanced Turbidimetric Immunoassay (PETIA) [11].

In our institution’s mixed ICU ward, the majority of cases were sepsis, with an incidence of about 48% and a mortality rate of 28% recorded in 2019 [12]. The causes of sepsis are mainly due to hospital-acquired pneumonia (50.28%), followed by community-acquired pneumonia (CAP) (20.3%), intra-abdominal infection (15.3%), necrotizing fasciitis (8.2%), urosepsis (3.4%), and meningitis (2.5%) [13]. Meropenem antibiotic was commonly prescribed in our ICU due to the majority of patients presenting with septic shock and high Acute Physiology and Chronic Health Evaluation II (APACHE II) and Sequential Organ Failure Assessment (SOFA) scores while waiting for culture [13]. The determination to use meropenem needs to be based on a balance between considerations of the severity of the sepsis and the inappropriate use of a broad-spectrum antibiotic that can lead to the emergence of a resistance organism. The normal value of PCT can be as low as less than 0.1 ng/mL and can rise up to 100 from baseline in a patient with sepsis [1]. Previous studies reveal PCT is a good biomarker for sepsis and its prediction or mortality [14,15,16,17,18].

The results of our study reveal the POC PCT tests exhibited comparable performance with the reference method, immunoassay performed in the laboratory. A small intercept, 0.02 (95% CI: −0.04 to 0.06) (Figure 3 and Figure 4), of Passing–Bablok analysis in our study, indicated minimal systematic differences between PCT measured at POC and automated immunoassay in the laboratory. A slope of 1.08, close to 1, indicates a strong linear relationship and proportional agreement between the methods (Figure 3 and Figure 4). Meanwhile, in our study, the Deming regression reveals an intercept of 0.43 (95% CI: −0.05 to 0.71) with a slope of 0.86 (95% CI: 0.78 to 1.15). Our study indicates that Finecare™ PCT exhibits minimally significant bias, 0.02 ng/mL, similar to results of other POC BRAHMS PCT studies by Kutz et al. in 2015, which reveal minimal bias, −0.02 ng/mL [19].

Based on the results, the mean plasma POC PCT value was lower in the sepsis population compared to the automated laboratory PCT. However, when excluding the higher values, the mean plasma POC PCT value was actually higher compared to the PCT assays performed in the laboratory. This finding is consistent with a previous study from 2021 [6].

The correlation coefficients were 0.98 for plasma and 0.96 for whole blood samples, which is similar to what has been reported in previous studies [20]. The precision was less than 5% for cut-offs of 0.5 and 2.5 ng/mL in our study, which is almost comparable to the reference immunoassay and AQT90 FLEX (Radiometer, Copenhagen, Denmark) [6]. Previous literature reported a CV of 9% for the 0.5 ng/mL cut-off and 11% for the 2.5 ng/mL cut-off value of PCT [6]. The best precision for POC was shown by the AQT90 FLEX (Radiometer, Copenhagen, Denmark) which revealed a CV of 3% for the 0.5 ng/mL cut-off and 2% for the 2.5 ng/mL cut-off value of PCT [6]. Meanwhile, other POC tests such as mLabs (Micropoint, Shenzhen, China) and Getein 1100 (Getein Biotech Inc., Nanjing, China) reveal CVs of 10% and 13% for the 0.5 ng/mL cut-off and 19% and 5% for the 2.5 ng/mL cut-off value of PCT [7]. Our study also reveals comparable findings compared to other immunoassays, such as in terms of inter- and intra-assay imprecision CV results, as follows: 2.0–3.8% and 3.2–9.5%, respectively, for Liaison BRAHMS PCT; 1.9–4.6% and 3.6–7.0%, respectively, for Vidas BRAHMS PCT; and 1.1–7.1% and 1.6–8.7%, respectively, for Roche BRAHMS PCT. The total imprecision for Siemens BRAHMS PCT is less than 11% [9]. Regarding the correlation coefficient, our study found an enhanced correlation of 0.98 when point-of-care PCT testing was performed using plasma samples rather than whole blood samples. This improved correlation contrasts with a previous study that reported a correlation of 0.96 when comparing whole blood point-of-care PCT samples to laboratory-based BRAHMS PCT testing [20]. In another study, the use of whole blood samples to test point-of-care PCT only provided a result of a 0.908 correlation when compared with the laboratory-based BRAHMS PCT testing [21]. For intensive care units equipped with bedside centrifuge machines, the use of plasma samples for point-of-care PCT testing is preferable. Although it requires an additional 10–15 min to centrifuge the sample and separate the cells from the plasma, this approach yields superior results compared to the use of whole blood samples for point-of-care PCT analysis.

The linearity found in the study is 0.995 compared to the reference assay, which was better than that reported from previous studies, which show fair to excellent correlation [6].

Since 2010–2019, several studies have been conducted to compare the accuracy of different methods of PCT measurement, with the highest bias reported by Lippi et al. in 2019 for Diazyme (Cobas) PCT, 24.7% and Maglumi PCT, 23.7% [22]. A total of 43 out of 60 comparison studies reveal a bias, with the intercept ranging between −0.2 and 0.2 ng/mL [14]. The bias was a significant finding for the lower cut-off values compared to the higher cut-off values of PCT.

## 5. Limitations

One limitation of the study is the inability to conduct all tests simultaneously due to an average of 10 samples per week being collected from sepsis patients in the intensive care unit for correlation study. However, for precision and linearity analysis, the test was performed on the same day. Out of the 40 samples analyzed, approximately 75% had values below 2 ng/mL. There were some outlier results; 97 ng/mL was measured on the BRAHMS PCT and 29 ng/mL was measured on the Finecare™ PCT, which needed to be removed. The percentage of outliers is 2.5%, which is smaller compared to the 5–6% reported in previous studies [19]. Clinically, these high-value results (97 ng/mL measured on BRAHMS PCT and 29 ng/mL measured on Finecare™ PCT) still indicate a similar severity of sepsis, as both are above the 10 ng/mL cut-off value. Another limitation of our study is that we did not measure the actual time taken for the laboratory to complete the PCT testing and make the results available to the clinician. Previous studies have reported an average time of about 144 min for laboratory PCT [19]. A larger sample size closer to the lower cut-off of the clinically significant value is necessary for future research on comparative studies of PCT [23]. The differences in results could also be due to differences in calibrators and antibodies [22].

## 6. Conclusions

Point-of-care testing of PCT using the Finecare™ PCT Rapid Quantitative Test on the Finecare™ FIA Meter III Plus demonstrates strong correlation, precision, and linearity compared to the laboratory immunoassay Elecsys^®^ BRAHMS Procalcitonin (PCT) performed on the Cobas e411, in patients with a diagnosis of sepsis admitted to the ICU.

## Figures and Tables

**Table 1:** Characteristics of the study participants (*n* = 41).

**Variables**		**Mean (SD)/** ***n*** **(%)**
Age (Years)		51.63 (16.55)
Sex		
	Female	14 (34.1)
	Male	27 (65.9)
BMI (Kg/m^2^)		26.44 (9.20)
AKI		
	No	28 (68.3)
	Yes	13 (31.7)
ESRF		
	No	37 (90.2)
	Yes	4 (9.8)
Total White Cell Count (×10^9^/L)		13.43 (4.85)
Temperature (°C)		36.74 (0.64)
Noradrenaline		
	No	25 (61.0)
	Yes	16 (39.0)
Ventilation		
	No	4 (9.8)
	Yes	37 (90.2)

All numerical variables were described as mean (SD), and all categorical variables were described as *n* (%), AKI = acute kidney injury, ESRF = end-stage renal failure.

**Table 2:** Procalcitonin (PCT) values of Elecsys^®^ BRAHMS and Finecare™.

Characteristic	*n* = 41 ^a^
Finecare™ PCT Rapid Quantitative Test (ng/mL)	4.46 (8.68)
Elecsys^®^ BRAHMS PCT (ng/mL)	4.67 (10.03)

^a^ Mean (SD).

**Table 3:** Paired *t*-test of plasma sample (*n* = 41).

Method	Mean (SD) ng/mL	Mean Different	t (df)	*p* Value
Finecare™ PCT Rapid Quantitative Test (ng/mL)	4.46 (8.68)	−0.21 (−0.91, 0.49)	−0.59 (39)	0.556
Elecsys^®^ BRAHMS PCT (ng/mL)	4.67 (10.03)			

## Data Availability

The data are available from the corresponding author upon request.
